# Supplementary material for: Suppressing microtubule detyrosination augments adeno-associated virus 2 endosomal escape and gene delivery
Source: J Cell Sci. 2025 Dec 5;138(23):jcs264190. doi: 10.1242/jcs.264190 (PMC12752493; doi:10.1242/jcs.264190)
Supplement: Supplementary information [file joces-138-264190-s1.pdf]

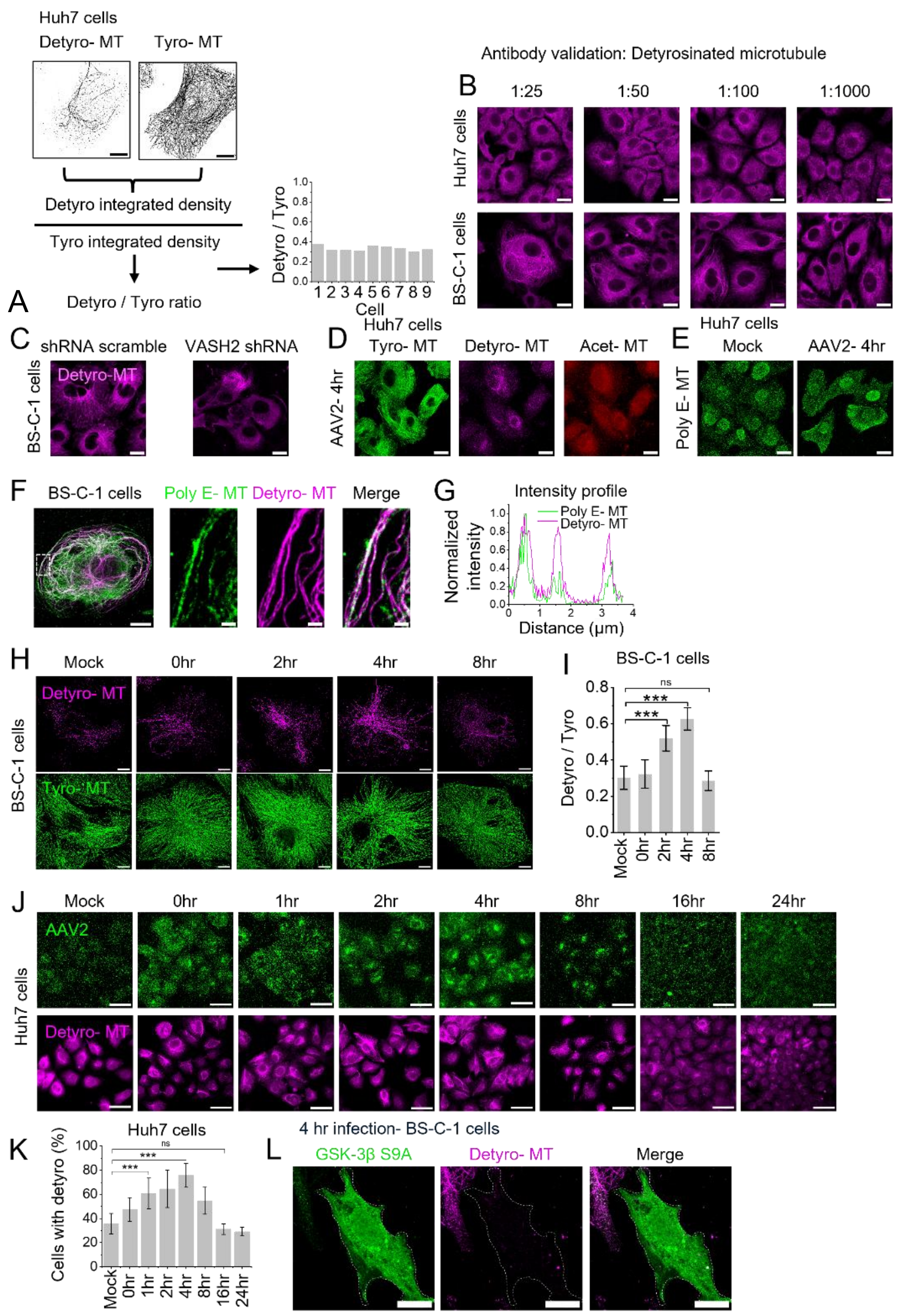

Fig. S1. Microtubule PTM quantification during AAV2 infection.

(A) SIM images with intensity threshold applied to deduce the integrated density and enumerate the ratio of detyrosinated to tyrosin ated microtubule represented by the bar plot. **(B)** Confocal imaging represents the detyrosinated microtubule antibody valid ation at different ratios (1:25, 1:50, 1:100 and 1:1000) in Huh7 and BS-C-1 cells. **(C)** Confocal images further validated the detyro sinated microtubule antibody specificity in the BS-C-1 cells treated with VASH shRNA versus scramble shRNA control. **(D-E)** Co nfocal microscopy of Huh7 cells infected with AAV2 for 4 hours and immunostained for tyrosinated (green), detyrosinated (magenta), and acetylated (red) microtubules (D). Note that Huh7 cells do not show detectable levels of acetylated microtubules as reported previously . Huh7 stained for polyglutamylated microtubule (Poly E) in mock vs AAV2 infected conditions (E). **(F-G)** BS-C-1 ce lls representing the colocalization of polyglutamylated and detyrosinated microtubules by the confocal image (F) and the intens ity profile analysis (G). (H-I) SIM images of BS-C-1 cells infected with AAV2 and fixed at 0-, 2-, 4-, and 8-hours post-infection show a change in the ratio of detyrosinated to tyrosinated microtubule (H). Quantification represents the of ratio of detyrosinated to tyrosin ated microtubules in BS-C-1 cells (I) analyzed from 10 images of three replicates. **(J-K)** Confocal imaging of mock and AAV2-infected Huh7 cells fixed at 0-, 1-, 2-, 4-, 8-, 16-, and 24-hours post-infection reveals an increase in the number of cells with microtubule detyrosination over time (J). Quantification of the percentage of Huh7 cells expressing detyrosinated microtubule at different time points post-infection (K) (n=12 cells from three independent replicates for each time point) highlights temporal modulation of microtubule post-translational modifications. **(L)** Suppression of detyrosinated microtubules observed in BSC-1 cells expressing GSK3β S9A, indicating that the signaling effect is not cell-type specific. Bars represent the mean value and whiskers represent the standard deviation. Statistical analysis via two paired two-sample t-tests confirmed significant differences (***$p<0.001$). Scale bars: (A, F, H, and L) 10 µm, (B, C, D and E) 20 µm, (J) 50 µm and (F subsets) 2 µm.

Journal of Cell Science • Supplementary information

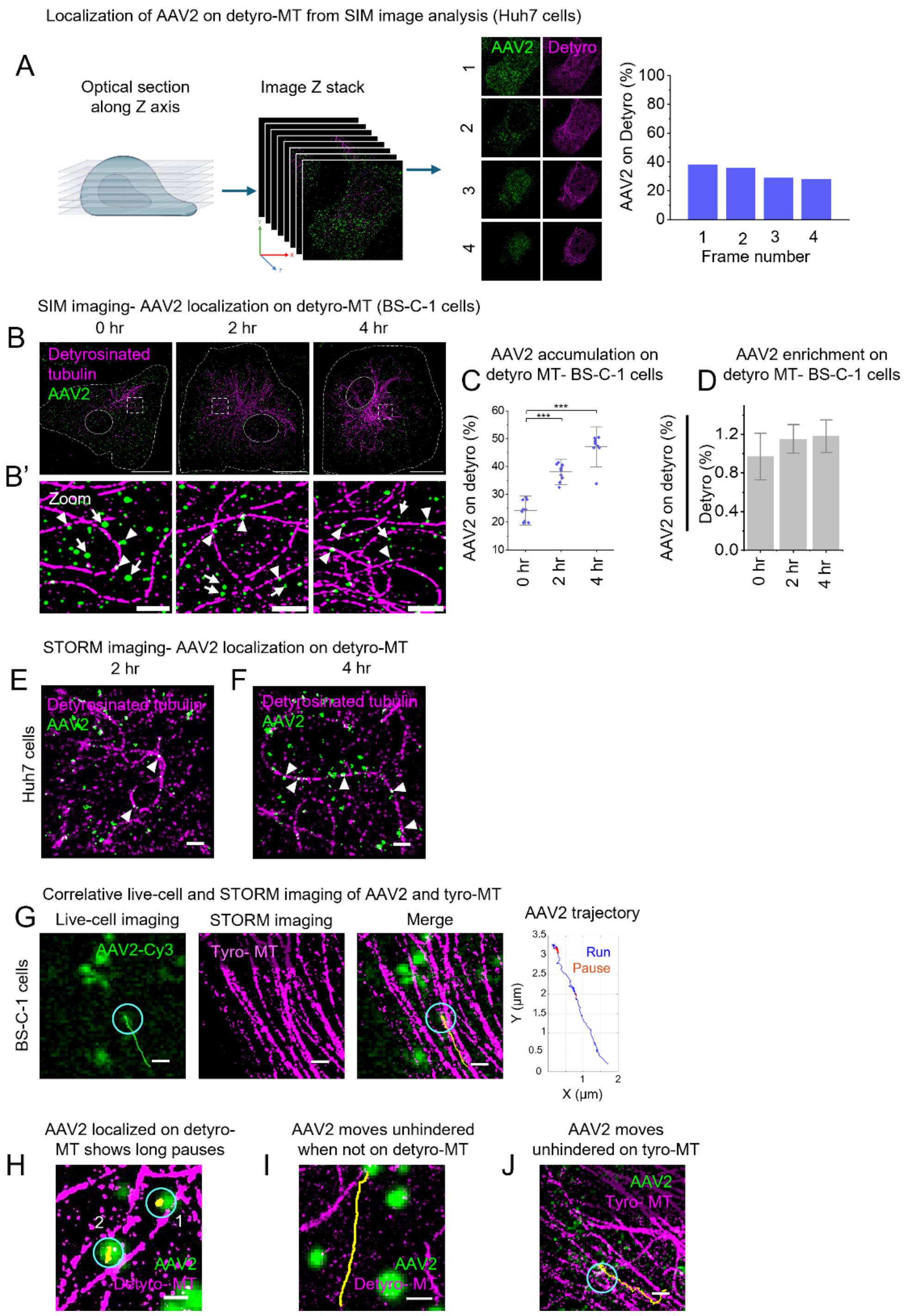

Fig. S2. Quantitation of AAV2 colocalization with detyrosinated microtubule.

(A) Sch ematic representation of optical sectioning and z-stack image acquisition for quantifying AAV2 localization on detyrosinated microtu bule, from a SIM z-stack image of AAV2 (green) and detyrosinated microtubule (magenta). From each Z-section, the percent age of AAV2 localized on detyrosinated microtubule was enumerated. **(B-D)** SIM images of BS-C-1 cells infected with AAV2 and fix ed at 0-, 2-, and 4-hours post-infection, indicating zoomed-in regions (B') from (B) highlight specific instances of AAV2 localization with arrowhead and arrows show AAV2 localized and not localized to detyrosinated microtubules, respectively. The graph shows the colocalization (C) and enrichment (D) of AAV2 on detyrosinated microtubules over time analyzed from 10 images nd three replicates. **(E-F)** STORM imaging of Huh7 cells infected with AAV2 shows the association of AAV2 (green) with detyrosinated microtu bule (magenta) at 2 hours (E) and 4 hours (F) post-infection, with arrows indicating AAV2 particles on detyrosinated microtu bule. **(G)** Correlative live-cell and STORM imaging of AAV2 and tyrosinated microtubules and its trajectory in BS-C-1 cells, demons trating the unhindered motility of AAV2 indicated in SI-video 4**. (H-J)** Images demonstrate the last frames of the SI-videos (video 2, 3 and 5) with overlay of AAV2 (green) tracks and STORM imaging of detyrosinated (H and I) and tyrosinated (J) microtubule (magenta). The dynamic behavior of AAV2 is captured in real-time (1-hour post-infection), with its trajectory overlaid on the video as a track (yellow), illustrating the path of viral particles as they interact with the microtubule- PTMs. Examples from the last frame of video show that the virus particles remain hindered on detyrosinated microtubule until the end of the recording (H). Directed transport of AAV2 along microtubule regions other than detyrosinated microtubule or when on tyrosinated microtubule remains unhindered, allowing faster movement (I and J). Scale bar: (B) 10 µm, (B') 2 µm and (E, F, G, H, I and J) 1 µm.

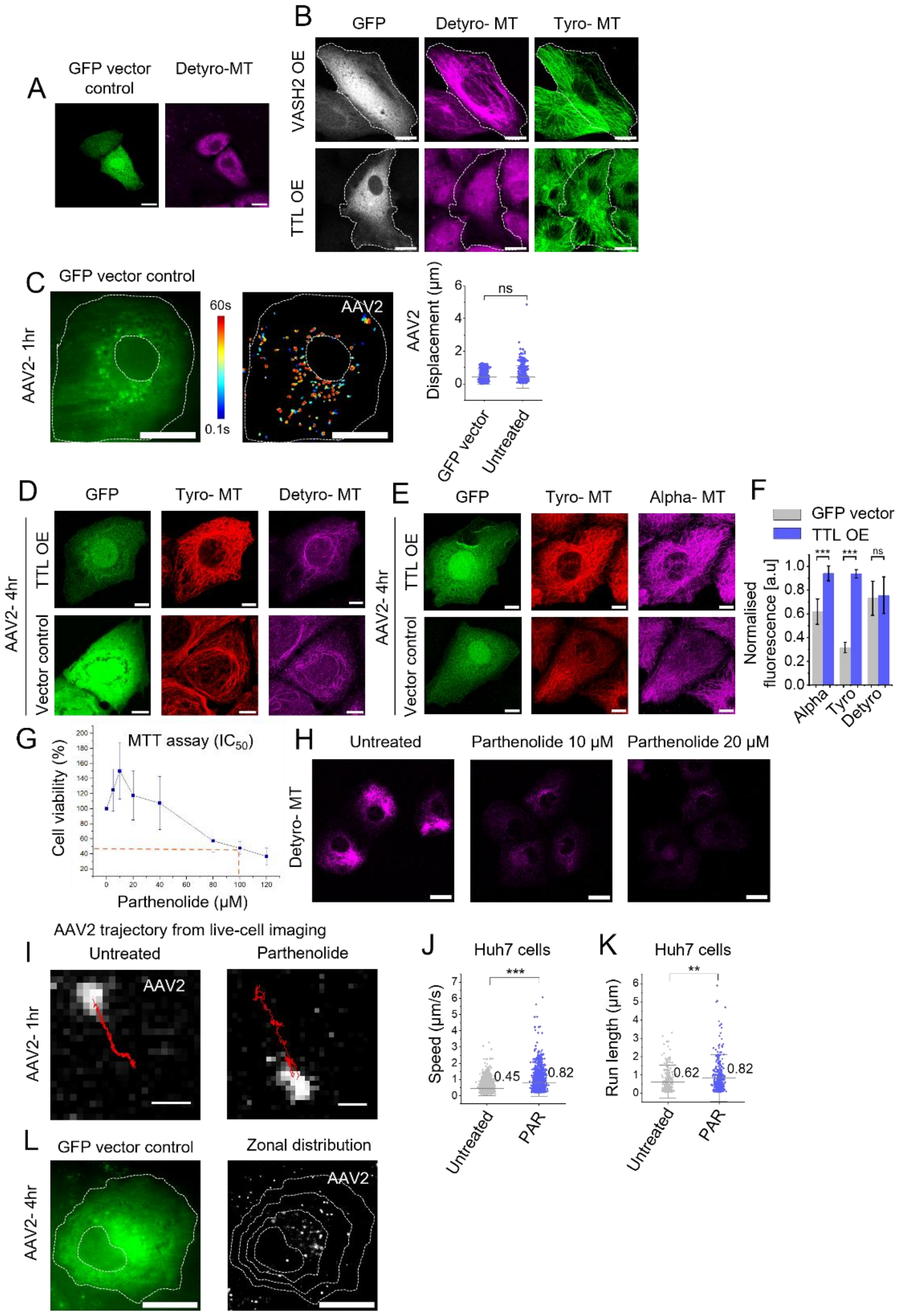

Fig. S3. Modulating detyrosination/tyrosination levels and their impact on AAV2 trafficking.

(A-B) C onfocal images of Huh7 cells expressing GFP vector control (A) or overexpressing the VASH2 or TTL with corresponding detyros inated and tyrosinated microtubule immunostaining (B). VASH overexpression shows a significantly high level of detyros inated microtubule in Huh7 cells and TTL overexpression upregulates microtubule tyrosination, in comparison to the GFP vector co ntrol that does not change the detyro levels. **(C)** AAV2 1hr live imaging in Huh7 cells expressing GFP vector control shows no significant difference with untreated and AAV2-infected cells. **(D-F)** Confocal images of Huh7 cells post 4hr infection represent that the TTL overexpression and tyrosinated, detyrosinated, and alpha microtubule immunostaining demonstrate that the detyrosinated microtu bule is unchanged (D) on TTL overexpression while tyrosinated and alpha tubulin (E) increase (F) as compared to the vector control. **(G)** Concentration-dependent cytotoxicity of parthenolide was assessed using an MTT assay, and IC_{50} was analyzed (IC_{50}= 100 µM). The graph shows mean values and standard deviation (n=3). **(H)** Imaging untreated and parthenolide-treated Huh7 cells (10 µM and 20 µM) infected with AAV2 reveals drug-induced suppression of detyrosinated microtubules. **(I)** Single-particle tracking of AAV2 in parthenolide-treated cells shows impaired viral motility compared to untreated control. **(J-K)** Graphs quantify AAV2 speed (J) and run length (K) under untreated and parthenolide-treated conditions, highlighting the effects of microtubule modification on viral dynamics. **(L)** The zonal distribution of AAV2 at 4 hours post-infection under GFP vector control conditions was comparable to that observed in untreated cells. Statistical analysis showing **p<0.005, ***p<0.001 and nonsignificant (ns). Scale bars: (A, B and H)20 µm, (I) 1 µm and (C, D, F and L) 10 µm. IC_{50}, half-maximal inhibitory concentration; MTT, 3-(4,5-dimethylthiazol-2-yl)-2,5-diphenyltetrazolium bromide.

Journal of Cell Science • Supplementary information

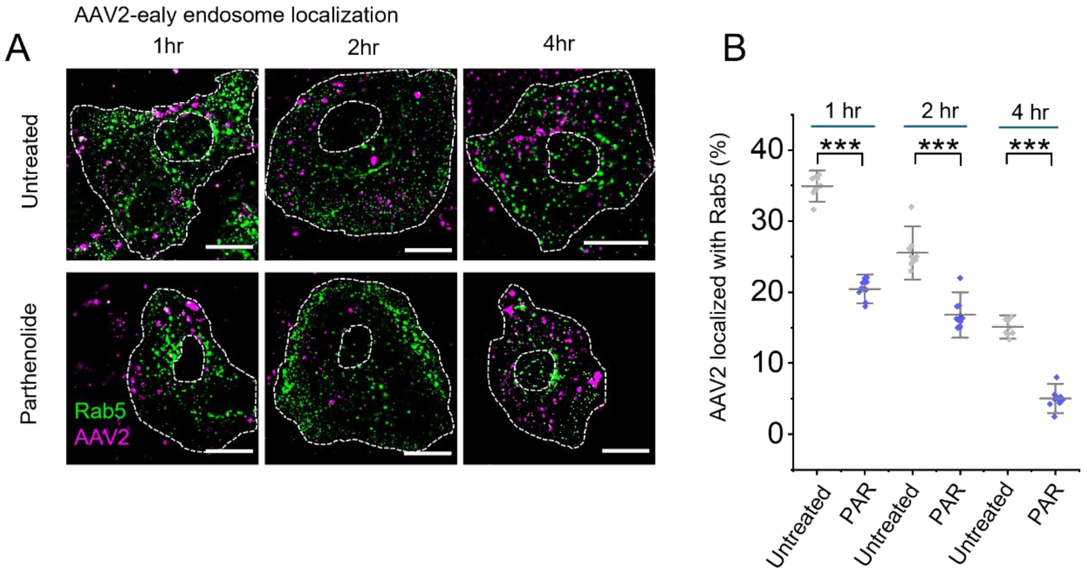

Fig. S4. Endosomal sorting of AAV2.
(A-B) Confocal imaging and Quantification of AAV2 (magenta) colocalization with early endosomes decorated by Rab5-GFP (green). Parthenolide-treated cells show significantly less colocalization of AAV2 with Rab5 compared to untreated controls (n=10 from three replicates) (B). In the graphs, the grey line indicates mean, and whiskers show the standard deviation from ten cells an alyzed (n=10). Statistical significance assessed via a two paired two-sample t-test. Significance indicated as ***p<0.001. Scale bars: (A) 20 µm.

Journal of Cell Science • Supplementary information

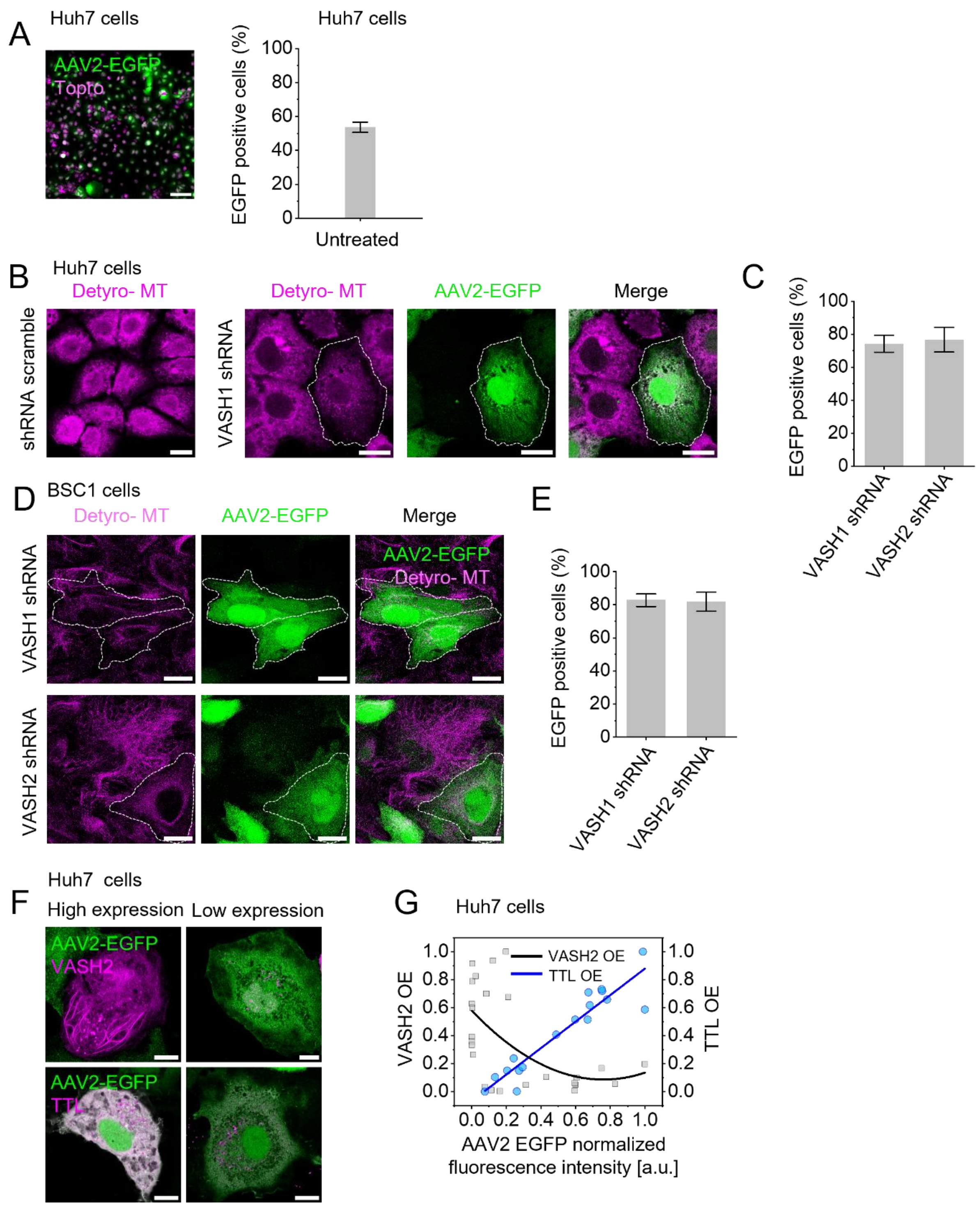

Fig. S5. VASH/ TTL overexpression (OE) and correlation with AAV2 transgene expression.
(A) Transduction of AAV2-EGFP (green) in Huh7 cells (nuclei marked with Topro: magenta), in untreated condition (no plasmids and drugs), shows ~55% transduction efficiency. **(B-C)** The image represents the detyrosinated microtubule staining (magenta) and AAV2 EGFP transduction (green) in Huh7 cells treated with VASH1 shRNA. The quantification represents the percentage of the cells transduced with AAV2 EGFP in VASH1/2 shRNA-treated cells. **(D-E)** AAV2-EGFP transduction in BS-C-1 cells treated with VASH1 and VASH2 (D) and quantification of the percentage of cells transduced (E). **(F-G)** Confocal imaging shows the effect of differential levels of VASH or TTL expression on AAV2-EGFP gene transduction (F) and their quantification (G). Scale bars: (A) 100 µm, (B and D) 20 µm, and (F) 10 µm.

Journal of Cell Science • Supplementary information

Blot transparency: Fig. 1E

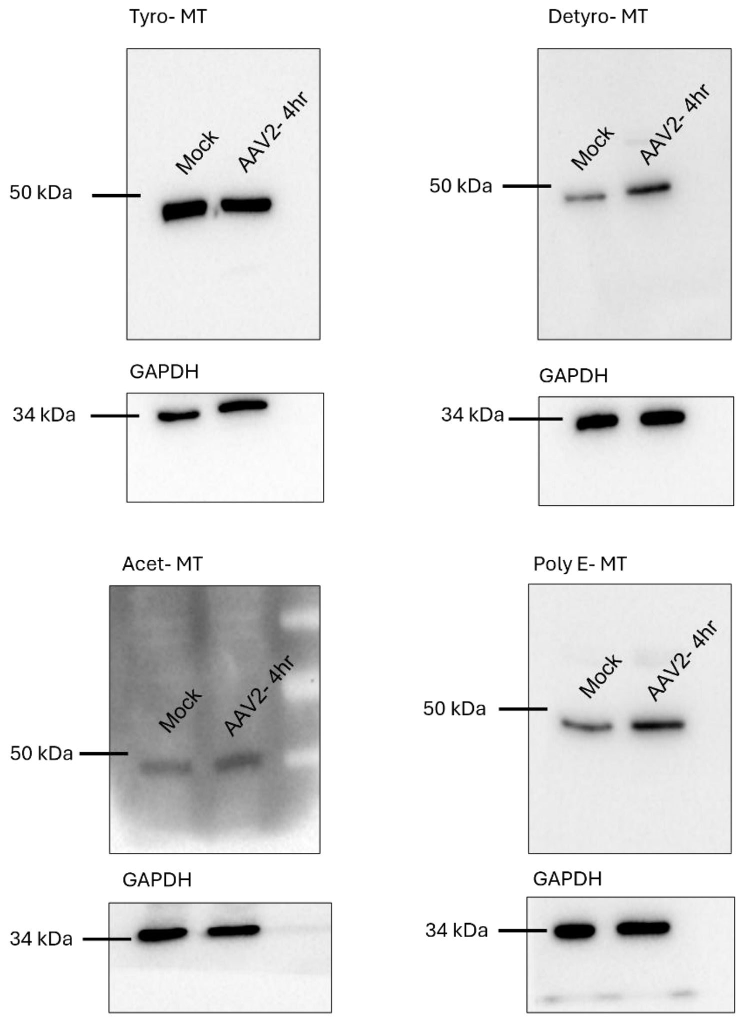

Fig. S6. Blot transparency.

Uncropped western blot images used in **Figure 1E** of the manuscript. The blots represent the different MT-PTMs and their respective GAPDH loading controls.

Table S1.

Antibody	Host species	Catalog number	Supplier
Alpha tubulin	Rabbit	ab18251	Abcam
Tyrosinated tubulin	Rat	ab6160	Abcam
Detyrosinated tubulin	Rabbit	ab48389	Abcam
Acetylated tubulin	Mouse	T6793	Sigma Aldrich
Tubulin polyglutamylation	Mouse	AB-20B-0020	Adipogen
AAV2	Mouse	10R-A110a	Fitzgerald
Phospho-GSK-3β (Ser9)	Rabbit	CST 5558	Cell signaling technology
CLASP2	Rabbit	PA5-109547	Invitrogen
GAPDH	Mouse	MA5-15738	Invitrogen
**Plasmids**	**Plasmid number**	**Supplier (repository)**	**Depositor**
pShuttle-mRFP-GSK3 S9A	24371	Addgene	Torsten Wittmann
pAd/CMV/V5-Clasp2α	89763	Addgene	Torsten Wittmann
pEGFP-CLASP2 512-650 9xS/A	24410	Addgene	Torsten Wittmann
Rab5-GFP			Kind gift from Indranil Banerjee (IISER Mohali, India)
Rab-GFP			
LAMP1-GFP			
VASH2-mCherry			Kind gift from Carsten Janke (Institut Curie, France)
VASH2 C158A mCherry			
TTL- Scarlet			
VASH-GFP			Kind gift from Minhaj Sirajuddin (InStem, Bengaluru, India),
TTL-GFP			

shRNA	TRC ID	Sequence	Supplier (repository)
VASH1	TRCN0000139334	CCGGCACAGGACATAGTGGTGCTT TCTCGAGAAAGCACCACTATGTCCT GTGTTTTTTG	shRNA consortium IISC Banglore, India
VASH2	TRCN0000163849	CCGGCAGGGACATGAGAATGAAGA TCTCGAGATCTTCATTCTCATGTCC CTGTTTTTTG	shRNA consortium IISC Banglore, India
Scramble	TRC1/1.5_SHC001-MISSION		Sigma Aldrich

Journal of Cell Science • Supplementary information

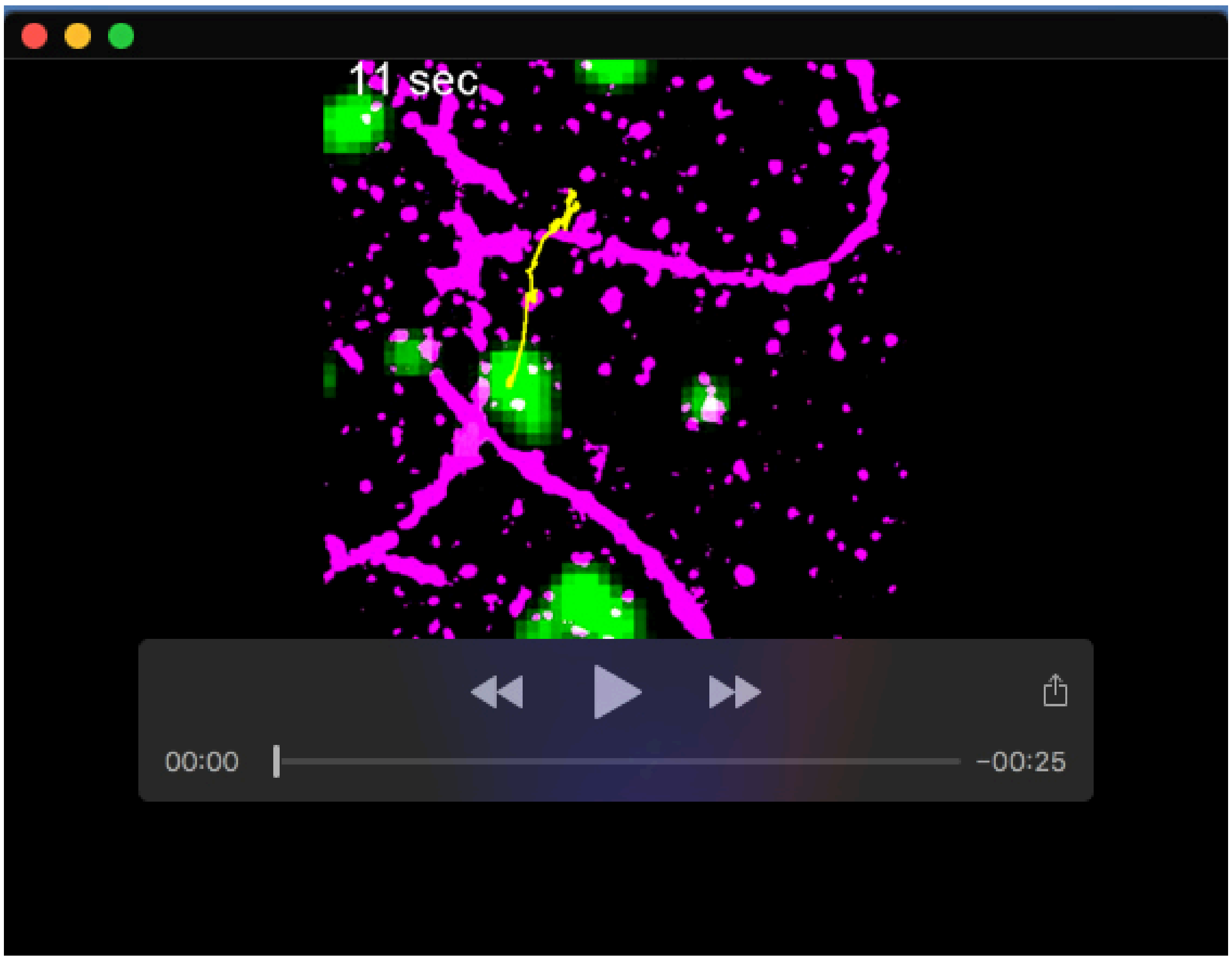

Movie 1. The video demonstrates the correlative imaging of AAV2 (green) in live-cell conditions and STORM imaging of detyrosinated tubulin (magenta). The real-time dynamics of AAV2 were observed one hour post-infection, with its trajectory displayed as a yellow track over the video, highlighting the movement of viral particles as they interact with the microtubule. AAV2 motility is notably restricted when encountering detyrosinated tubulin.

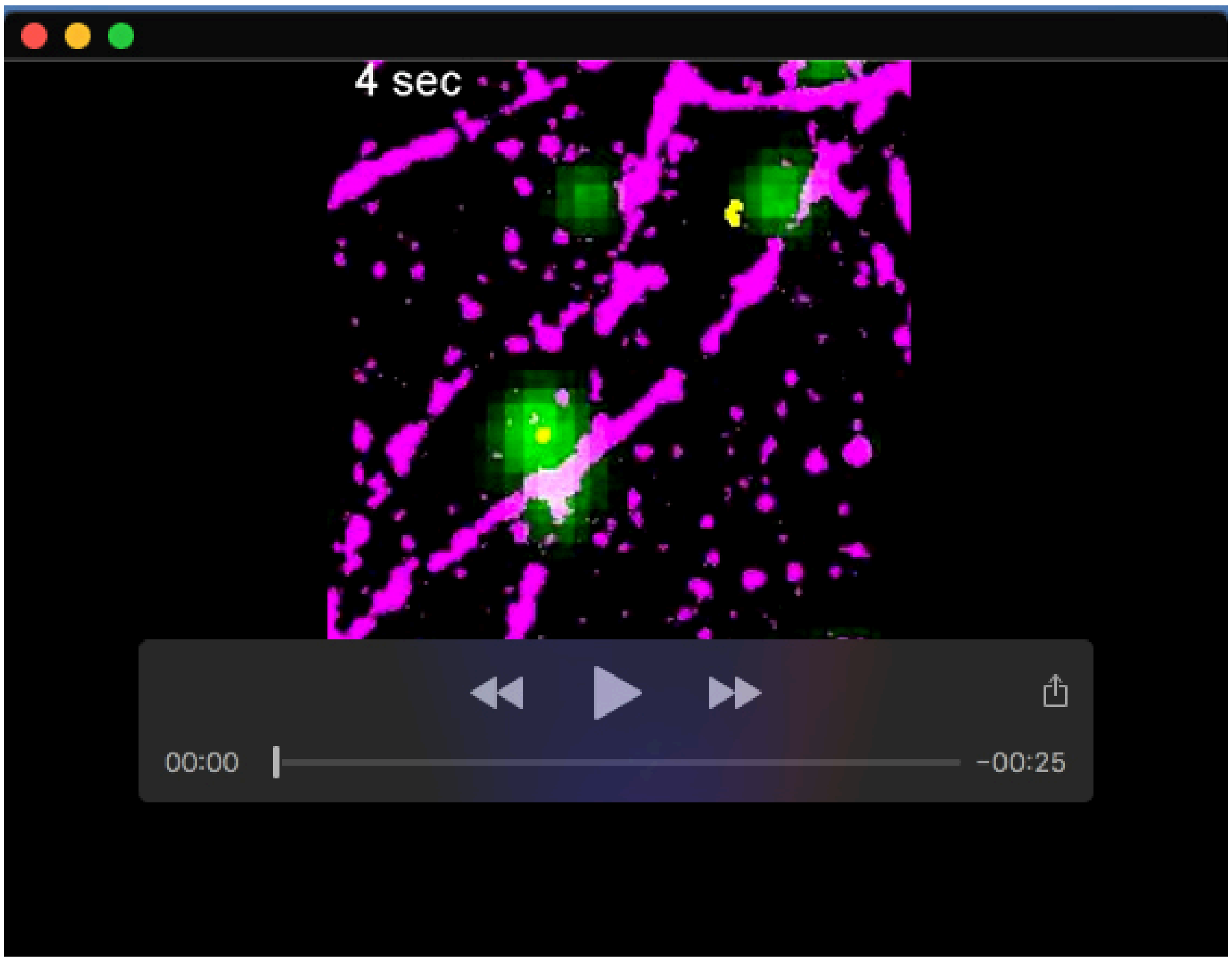

Movie 2. The majority of virus particles remain stalled on detyrosinated tubulin throughout the duration of the movie, highlighting its restrictive effect.

Journal of Cell Science • Supplementary information

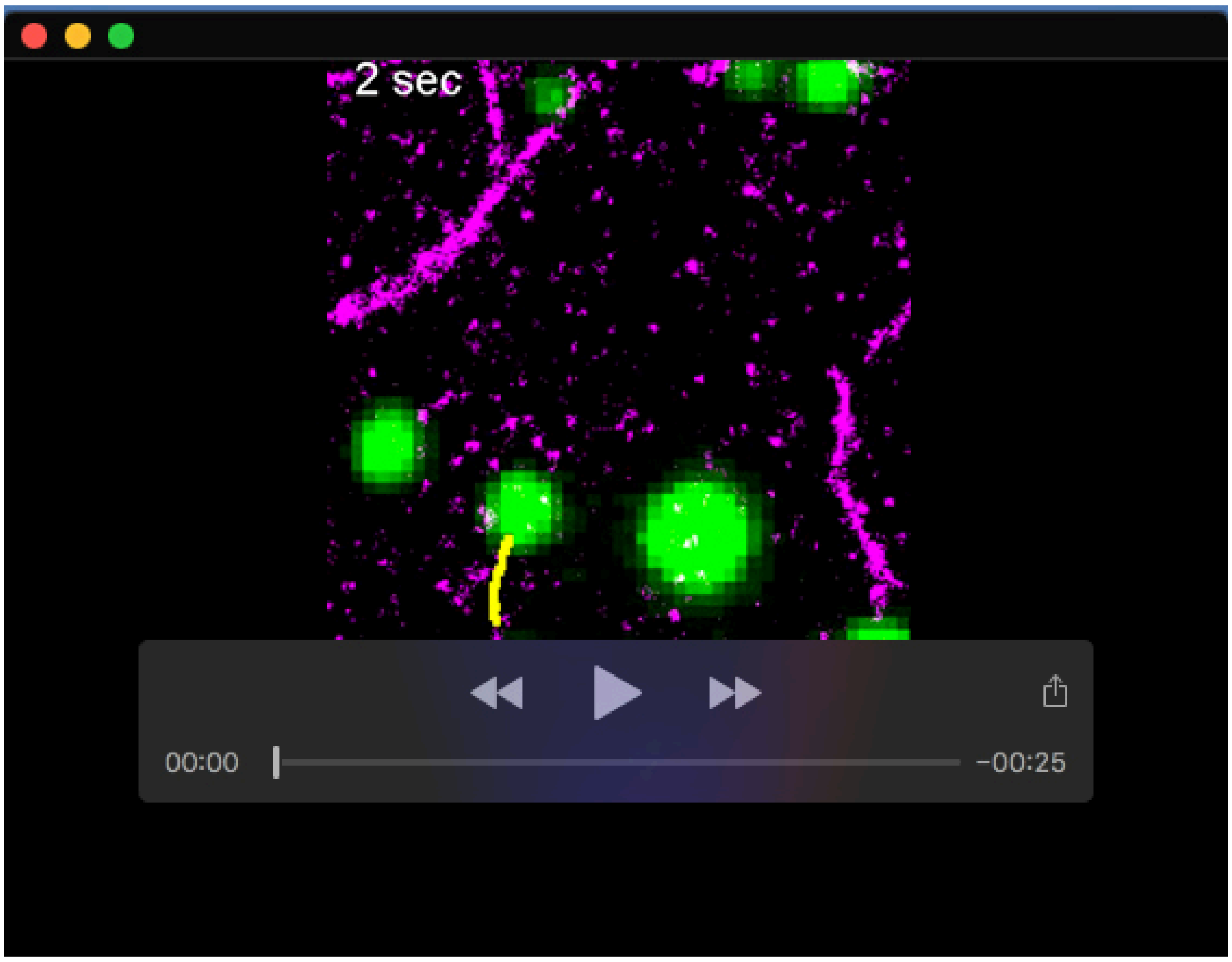

Movie 3. In contrast, when the virus moves along microtubule pathways that do not contain detyrosinated tubulin, its motility is unimpeded, allowing for faster movement.

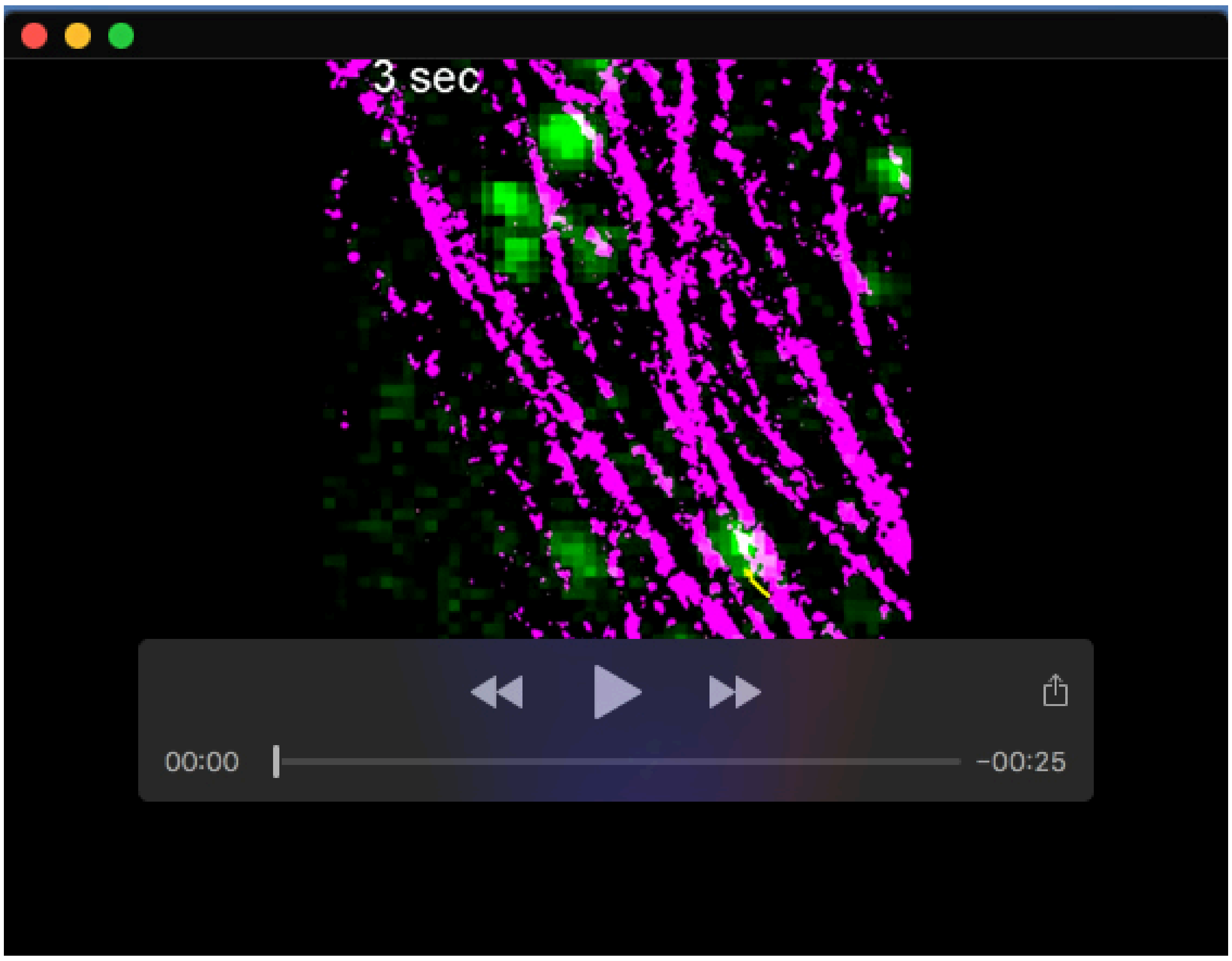

Movie 4. AAV2 trafficking on tyrosinated microtubule reveal that virus moves along the tyrosinated microtubule unimpeded.

Journal of Cell Science • Supplementary information

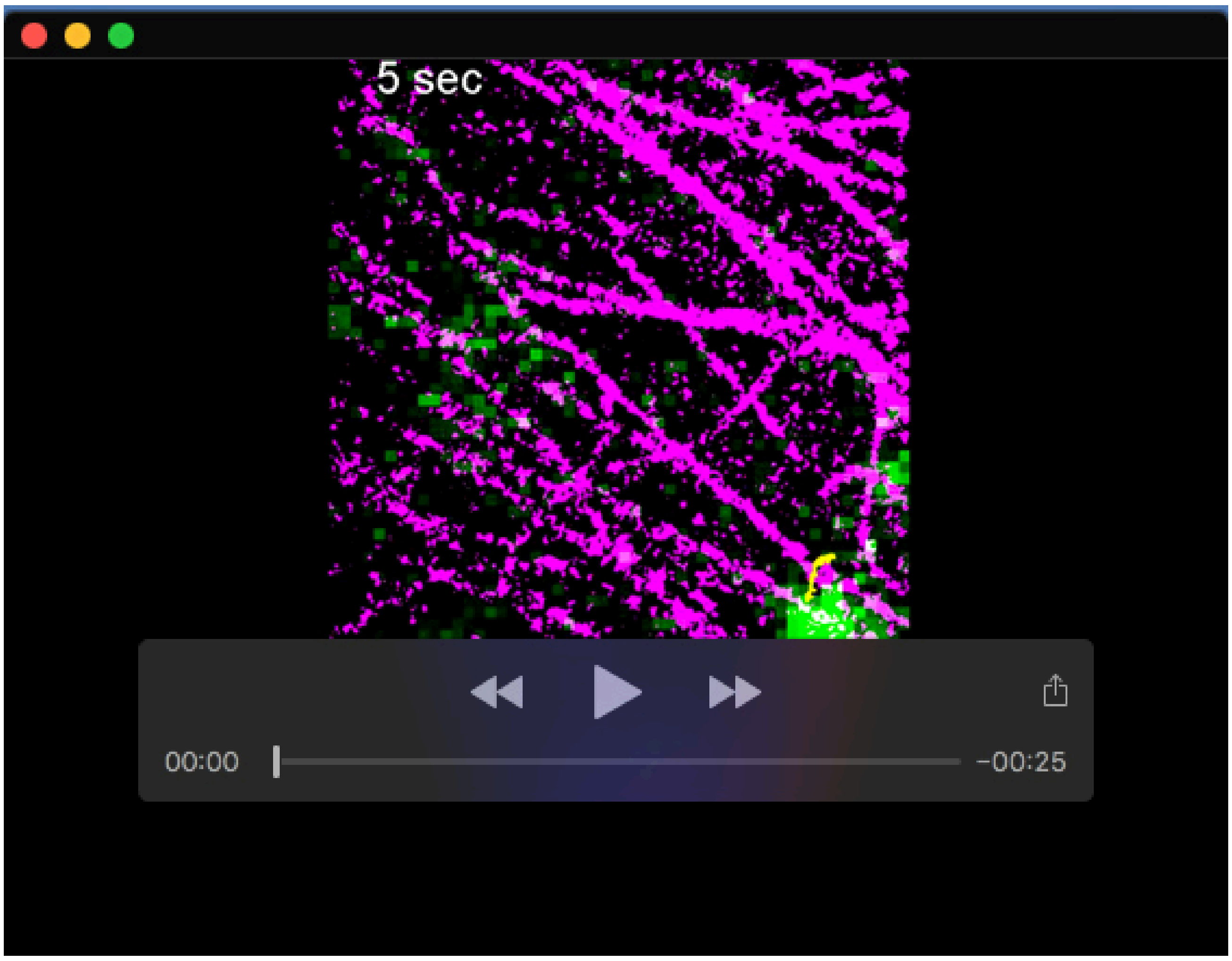

Movie 5. AAV2 trafficking on tyrosinated microtubule reveal that virus moves along the tyrosinated microtubule unimpeded.

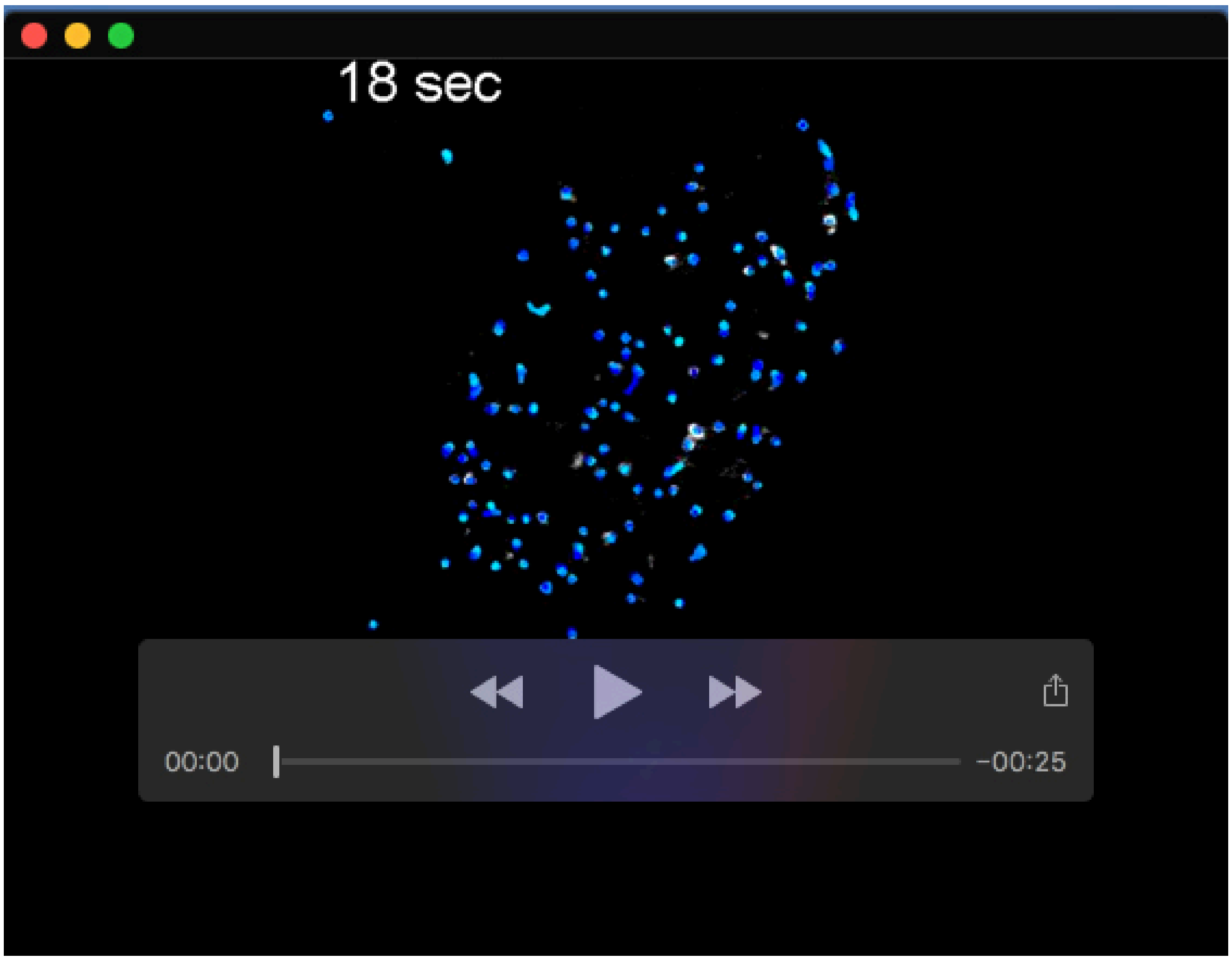

Movie 6. AAV2 Tracks derived from Trackmate analysis of Huh7 cells in control conditions.

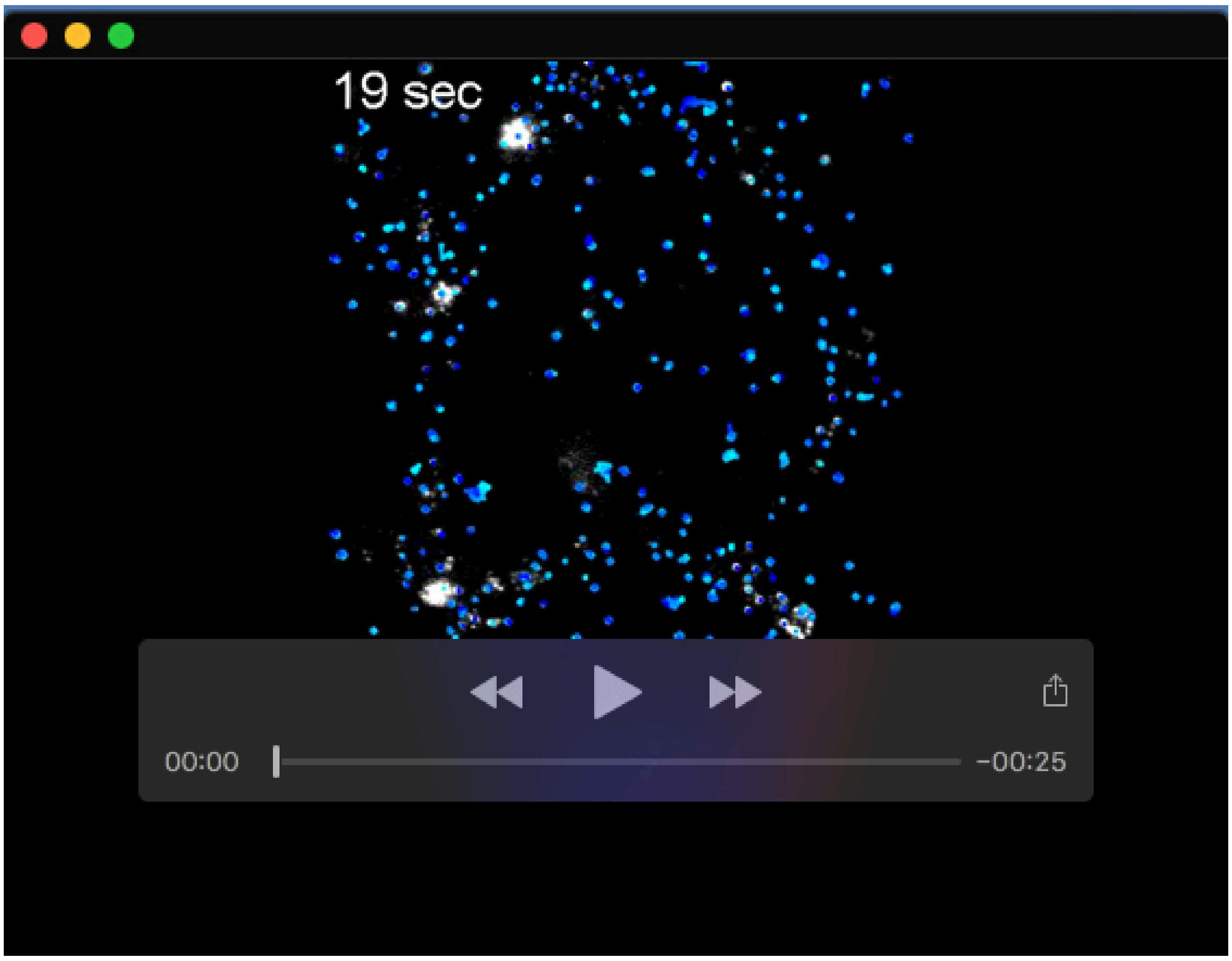

Movie 7. AAV2 Tracks in Huh7 cells overexpressing VASH2.

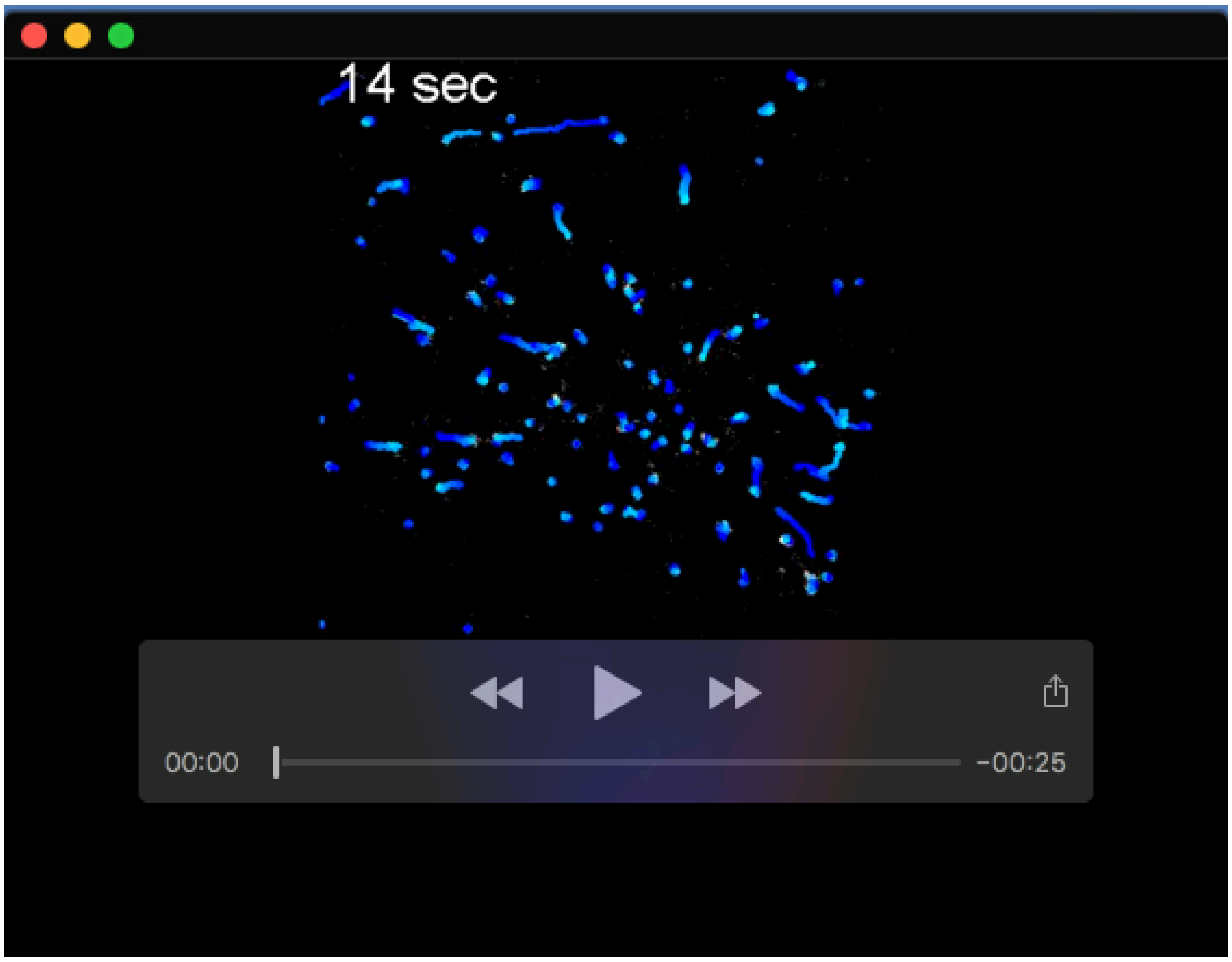

Movie 8. AAV2 Tracks in Huh7 cells overexpressing TTL.

Journal of Cell Science • Supplementary information

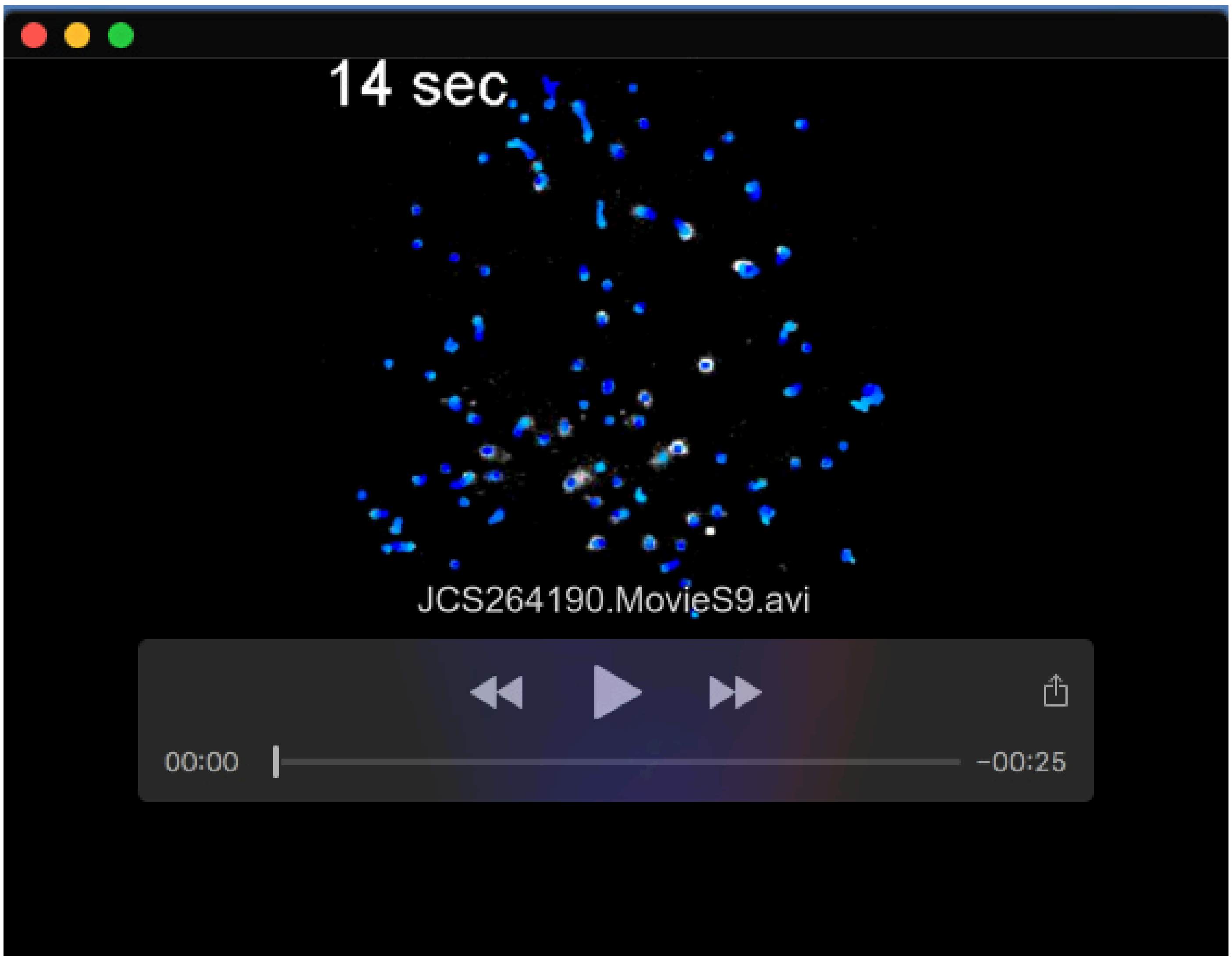

Movie 9. AAV2 Tracks in Huh7 cells pretreated with parthenolide.

Journal of Cell Science • Supplementary information
